# Luteolin suppresses inflammation and oxidative stress in chronic obstructive pulmonary disease through inhibition of the NOX4‐mediated NF‐κB signaling pathway

**DOI:** 10.1002/iid3.820

**Published:** 2023-04-27

**Authors:** Mingfei Li, Huifang Wang, Yun Lu, Jinwen Cai

**Affiliations:** ^1^ Emergency Department Hospital of Chengdu University of Traditional Chinese Medicine Chengdu China; ^2^ Department of Respiratory and Critical Care The Third Xiangya Hospital of Central South University Changsha China

**Keywords:** chronic obstructive pulmonary disease, inflammation, luteolin, NF‐κB pathway, NOX4, oxidative stress

## Abstract

**Introduction:**

Chronic obstructive pulmonary disease (COPD) is associated with chronic inflammation that predominantly affects the lung and peripheral airways. Previous investigation has underlined the efficacy of luteolin in the treatment of inflammation‐related symptoms. Accordingly, our study concentrates on unveiling the effect of luteolin on COPD.

**Methods:**

Mice or A549 cells were treated with cigarette smoke (CS) to establish COPD models in vivo and in vitro. Then, the serum and bronchoalveolar lavage fluid of mice were harvested. The lung tissues of mice were subjected to hematoxylin‐eosin staining to observe the degree of damage. The inflammation and oxidative stress factors level were calculated via enzyme‐linked immunosorbent assay and quantitative real‐time polymerase chain reaction. The expressions of nuclear factor‐kappa B (NF‐κB) pathway‐related factors were detected by Western blot.

**Results:**

In in vivo experiments, CS treatment reduced the weight of mice and promoted lung tissue damage, while luteolin attenuated the effect of CS on the mice. Moreover, luteolin inhibited the inflammation factors level, oxidative stress, and NADPH oxidase 4 (NOX4)‐mediated NF‐κB signaling pathway in CS‐induced COPD mice. Similar results were obtained in in vitro experiments that luteolin alleviated CS‐induced inflammation, oxidative stress, and NOX4‐mediated NF‐κB signaling pathway activation in CS‐treated A549 cells. Besides, NOX4 overexpression offset the impacts of luteolin on the CS‐induced A549 cells.

**Conclusion:**

Luteolin alleviates inflammation and oxidative stress in COPD via NOX4‐mediated NF‐κB signaling pathway, which provides a theoretical basis for the treatment of COPD with luteolin.

## Graphical abstract

Luteolin inhibited the inflammation and oxidative stress in cigarette smoke (CS)‐treated mice. Luteolin alleviated inflammation and oxidative stress in CS‐treated cells. Luteolin targeted and downregulated the NADPH oxidase 4 (NOX4) in CS‐treated mice and cells. Luteolin alleviated inflammation and oxidative stress in chronic obstructive pulmonary disease via NOX4‐mediated nuclear factor‐kappa B signaling.

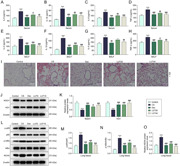

## 1 INTRODUCTION

Currently, chronic obstructive pulmonary disease (COPD) is a common lethal factor in our modern life, with the elderly as the primary victim.^1^, ^2^ The symptoms of COPD include cough and dyspnea, and the trigger involves significant exposure to noxious particles or gases, especially cigarette smoke (CS) which has been recognized as the leading cause.^3^, ^4^, ^5^ CS continuously induces airway inflammation through complex signaling pathways and produces reactive oxygen species (ROS), interleukins (IL), chemokines, and proteases via direct or indirect stimulation of airway epithelial cells and macrophages.^6^, ^7^ The elevation of inflammation indexes results in chronic airway inflammation and structural alterations, causing the loss of lung function.^8^ Additionally, exogenous oxidants in CS and air pollution bring about oxidative stress in COPD, which leads to the endogenous generation of ROS by inflammatory and structural cells within the lung.^9^ Oxidative stress can exacerbate chronic inflammation, stimulate fibrosis and emphysema, cause corticosteroid resistance, accelerate lung aging, induce DNA damage, and provoke the formation of autoantibodies.^9^ Thus, the suppression of inflammatory responses and antioxidative stress are considered important treatment strategies for inhibiting the development of COPD.

NADPH oxidase 4 (NOX4) is not only a member of the NOXs enzyme family that produces ROS, but also a major source of ROS.^10^ Also, NOX4 has been proven to be involved in the occurrence and development of various diseases including COPD, where NOX4 expression is both upregulated in epithelial cells and small airway smooth muscle cells of the COPD model and is positively correlated with the severity of chronic obstructive pulmonary airflow.^11^ In addition, luteolin can suppress smooth muscle cell proliferation by reducing NOX4 production.^12^ More importantly, based on the data retrieved from AutoDock software, we discovered that NOX4 can bind with luteolin. Accordingly, we wondered if luteolin can target NOX4 to exert its effects on COPD.

Luteolin, one of the flavonoids, is widely present in most vegetables, fruits, and herbs, such as thyme and green pepper.^13^, ^14^, ^15^ As the main component of trichosanthis, which has been identified to have an antioxidant effect, luteolin is used as an anti‐inflammatory drug, owing to its own pharmacological activity against inflammation.^16^, ^17^, ^18^ In lung injury, luteolin suppresses the levels of proinflammatory cytokines, like IL‐6 and IL‐1β, and attenuates the oxidative stress in septic mice.^19^ In addition, luteolin has been proven to promote mucociliary clearance in COPD, and mucus production has been revealed as a characteristic of COPD.^20^ However, the specific effect of luteolin on COPD remains unclear and requires further study. Based on this, we used luteolin as the agent to fathom its possible role in the treatment of COPD.

Furthermore, an existing study has emphasized that luteolin can protect against diabetic cardiomyopathy via nuclear factor‐kappa B (NF‐κB) pathway‐mediated inflammation, along with the activation of antioxidant responses.^21^ NF‐κB signaling has been recognized as a typical proinflammatory signaling pathway, based on its effects on the expressions of proinflammatory genes, possibly through the activation of IL‐1 and tumor necrosis factor‐α (TNF‐α).^22^, ^23^ Also, the overexpression of receptors for advanced glycation end‐products perpetuates oxidative stress and leads to nervous inflammation via NF‐κB signaling.^24^ Moreover, the NF‐κB signaling pathway is the key factor of acute lung injury and inflammation, and runt‐related transcription factor 1 can regulate inflammation response via inhibiting NF‐κB signaling.^25^, ^26^ Previous experiments also proved that curcumin can inhibit CS‐induced inflammation by modulating the peroxisome proliferator‐activated receptor gamma‐NF‐κB signaling pathway in vitro and in vivo.^27^ Besides, luteolin ameliorates TNF‐α‐induced oxidative stress and inflammation in human umbilical vein endothelial cells (HUVECs) by suppressing the NOX4/ROS‐NF‐κB pathway.^28^ Given the above information, we hypothesized that luteolin may also participate in the progression of COPD by regulating the NOX4‐mediated NF‐κB signaling pathway.

In our present study, we aimed to discover the role of luteolin in COPD and to determine whether the effects of luteolin on COPD are associated with the regulation of NOX4‐mediated NF‐κB signaling in vitro and in vivo, with the hope to provide a theoretical basis for the treatment of COPD.

## 2 MATERIALS AND METHODS

### 2.1 Animal COPD model construction and treatment

Male BALB/c mice (*n* = 30, 6–8 weeks old, 20–25 g) were obtained from SLAC Laboratory Animal Center (D011.10), and the mouse model of COPD was established as previously described.^29^, ^30^

In detail, the mice were divided into five groups, with six mice in each group: Control group, CS group, dexamethasone (Dex) group, luteolin (LUT 20 and 40) groups. The COPD model was established as follows: the mice were exposed first to 100 mg/m^3^ CS for 15 days, and then to 250 mg/m^3^ CS for 5 days/week for 75 consecutive days (6 h/day).

The mice in the Control group were normally fed, while those in the CS group were subjected to the construction of the COPD model as mentioned above. In addition, the mice in the Dex group, the LUT 20 group, and the LUT 40 group also underwent the construction of a COPD model, and during their exposure to CS at 250 mg/m^3^ for a total of 75 days, mice were treated with 2 mg/kg Dex (ST1258; Beyotime), 20 mg/kg LUT (L409168; Aladdin), or 40 mg/kg LUT by gavage for 75 days.

After 90 days of treatment, the mice were weighed by an electronic balance (E0298; Beyotime), followed by being anesthetized using 40 mg pentobarbital sodium (P3761; Sigma‐Aldrich) and killed by cervical dislocation.^31^ Blood samples were collected from the abdominal aorta and then centrifuged at 4500 rpm for 15 min. The serum supernatant fluids were collected and stored at −80°C for tests of inflammatory factors and enzyme activity. To collect bronchoalveolar lavage fluid (BALF), the trachea was inserted into the lungs of mice, and then the lungs were lavaged three times with 3 mL saline solution, and the BALF was collected and stored at −80°C. The lung tissue was harvested for staining assays.

### 2.2 Hematoxylin‐eosin (H&E) staining

For the histological examination, the paraffin‐embedded section (5 μm thickness) was prepared. A commercial H&E staining kit (C0105M; Beyotime) was used to examine the damage to lung tissue from mice in each group.^32^ The paraffin‐embedded sections were dewaxed in xylene (X112054; Aladdin) for 5 min twice. Then the sections were washed with both gradient concentrations of ethanol (100%, 90%, 80%, and 70%, E111991; Aladdin) and distilled water for 2 min. After that, the sections were stained with hematoxylin staining solution for 10 min and washed with water for 10 min, following which the sections were colored with eosin staining solution for 2 min. The sections were dehydrated using ethanol (70%, 85%, 95%, and 100%). Tissue sections were additionally treated with xylene and sealed with neutral gum (N116470; Aladdin). The THUNDER Imager Tissue Panoramic tissue microscopic imaging system (Leica) was finally used to observe the results of the staining with the indicated magnification at ×100.

### 2.3 Cell culture

Human alveolar epithelial cell line A549 (CRM‐CCL‐185; American type culture collection) was cultured in the Dulbecco's Modified Eagle Medium (DMEM, 11965092; Thermo Fisher Scientific) supplemented with 10% fetal bovine serum (FBS, 10099141; Thermo Fisher Scientific) and 1% penicillin–streptomycin (C0222; Beyotime)^33^ in an incubator (ICA175; IRM Technology GmbH) at 37°C with 5% CO_2_.

After cells were grown to be 70% confluent, Trypsin‐EDTA Solution (C0201; Beyotime) was used to digest cells for 3 min. Next, trypsin was removed, and the cells were cultured in a new medium and were subcultured every 3 days at a ratio of 1:3.

### 2.4 Cell transfection or treatment

For the transfection, the NOX4 overexpression plasmid and the negative control (NC) plasmid were purchased from GenePharma. A549 cells were cultured in a six‐well plate until the cell density reached approximately 80%, the plasmids above were transfected into the cells for 48 h with the aid of the Lipo8000 transfection reagent (C0533; Beyotime).

After transfection, the cells received the construction of a cell COPD model or the treatment of drugs. The construction of the COPD model was based on the previous study.^34^ Specifically, cigarette smoke extract (CSE) was first prepared on the day of the experiment, when the cigarette filter was cut and the smoke from two cigarettes was bubbled through the 25 mL serum‐free cell medium using a vacuum pump. The absorbance of 100%, 20%, 10%, 5%, 2.5%, and 1.25% CSE was determined at 320 nm using a spectrophotometer, and the resulting 100% CSE was standardized.

For the modeling, A549 cells with the transfection or not were treated with 1% concentrations of CSE for 24 h. For drug treatment, A549 cells after the transfection or CSE exposure were treated with either 1 μΜ Dex or at LUT 100 or 200 μΜ for 24 h, respectively.^35^, ^36^

### 2.5 Cell counting kit‐8 (CCK‐8) assay

The optimal concentration(s) of luteolin used in this research was determined based on the data of the CCK‐8 assay. In brief, A549 cells were cultured in 96‐well plates (1000 cells/well) for 12 h, after which 10, 100, 200, and 400 μΜ luteolin was added into the 96‐well plates to incubate with the cells for 48 h. Then, 10 μΜ CCK‐8 solution (C0037; Beyotime) was further added to incubate with the cells for 1 h. Finally, to determine the viability, the optical density (OD) value of the cells was determined by a microplate reader (Varioskan LUX; Thermo Fisher Scientific) at the wavelength of 450 nm.

### 2.6 Enzyme‐linked immunosorbent assay (ELISA)

Mouse IL‐1β ELISA kit (Ek‐M20166; EK‐Bioscience), mouse IL‐6 ELISA kit (Ek‐M20193; EK‐Bioscience), mouse IL‐8 ELISA kit (Ek‐M21259; EK‐Bioscience), mouse TNF‐α ELISA kit (Ek‐M21159; EK‐Bioscience), mouse superoxide dismutase (SOD) ELISA kit (Ek‐M21269; EK‐Bioscience), mouse catalase (CAT) ELISA kit (Ek‐M20464; EK‐Bioscience), mouse malondialdehyde (MDA) ELISA kit (Ek‐M21268; EK‐Bioscience), human SOD ELISA kit (EK‐H10482; EK‐Bioscience), human CAT ELISA kit (EK‐H10849; EK‐Bioscience), and human MDA ELISA kit (EH4174; FineTest) were used in this research as per the manufacturer's instructions.

Briefly, the serum and BALF of mice or the cell medium were added to the reaction well, followed by the addition of 50 μL biotin‐labeled antibody to the well immediately, and incubated at 37°C for 1 h. Subsequently, the liquid was removed, and the sample was rinsed with a washing solution for 30 s three times. Eighty microliters of affinity streptomycin‐HRP was added and cultured with the sample at 37°C for 30 min, after which the washing step was repeated three times. Next, the sample was additionally incubated at 37°C for 10 min in the dark with 100 μL substrate. The mixture was mixed and color‐developed at 37°C for 10 min. Finally, 50 μL stop buffer was added to terminate the reaction. A microplate reader was used to determine the OD value of each well, and the concentration of the factors above was calculated following the manufacturer's instructions.

### 2.7 Quantitative real‐time polymerase chain reaction (qRT‐PCR)

TRIzol™ Reagent (15596‐026; Invitrogen) was employed to isolate the total RNA from the cultured A549 cells. In detail, 1 mL Trizol was mixed with the cells and incubated for 2 min. The solution was subsequently added into the 1 mL Eppendorf tubes (EP), and then co‐centrifuged with 200 μL chloroform (C2432; Sigma‐Aldrich) at 4000*g* for 15 min (4°C) using a centrifuge (75002560; Thermo Fisher Scientific). The liquid of the aqueous phase was collected, and 500 μL isopropanol (I141145; Aladdin) was added to the EP tube for a 10 min mixture. The precipitated RNA was harvested after another centrifugation at 12,000*g* for 10 min (4°C). Following the washing with 1 mL 75% ethanol, which was configured with 100% ethanol (PHR1373; Merck) and DEPC‐ddH_2_O (SJ01‐A; DMD BioMed), the RNA precipitates were collected by another centrifugation at 12,000*g* for 5 min (4°C). The collected RNA precipitates were finally dissolved in 30 μL RNA‐free H_2_O (10977023; Thermo Fisher Scientific), and the concentration of the harvested RNA was measured in a spectrophotometer (DR6000; HACH).

PrimeScript RT Master Mix (RR036A‐1; TAKARA) was applied for the reverse transcription of RNA into cDNA. Subsequently, the PCR was conducted at the indicated thermal cycles as per the instructions. To carry out the quantification test, the $2^{‐\Delta \Delta C_{t}}$ method was used to calculate the relative mRNA expression levels, with β‐actin as the housekeeping control.^37^ The primer sequences of NOX4, IL‐6, IL‐1β, and TNF‐α were as follows: NOX4, 5′‐AGATGTTGGGGCTAGGATTG‐3′ (forward), 5′‐TCTCCTGCTTGGAACCTTCT‐3′ (reverse); IL‐6, 5′‐ACTCACCTCTTCAGAACGAATTG‐3′ (forward), 5′‐CCATCTTTGGAAGGTTCAGGTTG‐3′ (reverse); IL‐1β, 5′‐ATGATGGCTTATTACAGTGGCAA‐3′ (forward), 5′‐GTCGGAGATTCGTAGCTGGA‐3′ (reverse); TNF‐α, 5′‐CCTCTCTCTAATCAGCCCTCTG‐3′ (forward), 5′‐GAGGACCTGGGAGTAGATGAG‐3′ (reverse); β‐actin, 5′‐CATGTACGTTGCTATCCAGGC‐3′ (forward), 5′‐CTCCTTAATGTCACGCACGAT‐3′ (reverse).

### 2.8 Western blot assay

This assay was carried out with the materials and reagents ordered from Beyotime unless specified. The protein in the lung tissues of mice was extracted by T‐PER tissue protein extraction reagent (78510; Thermo Fisher Scientific), and that in A549 cells was isolated using RIPA Lysis Buffer (P0013C) containing protease inhibitor cocktail (P1011). The concentration of the harvested protein sample was quantified using the BCA protein quantitation assay kit (P0012S). Twenty microliters of prepared protein sample liquid was loaded separately on SDS‐PAGE (P0012AC), and transferred to the PVDF membrane (FFP33). The membrane was then blocked by skim milk and incubated with primary antibodies at 4°C overnight, followed by washing with Tris‐Buffered Saline with Tween20 (TBST, ST673) on a shaker at room temperature thrice, with 5 min for each time. After that, the membrane was incubated with the secondary antibodies at room temperature for 1 h, and then washed with TBST for 10 min. Finally, the chemiluminescent reagent (P0018FS) was incubated with the membrane for detecting the protein bands under the gel imaging system (12012165; Bio‐Rad).

The primary antibodies used here were those against phosphorylated IκBα (p‐IκBα; #2859, 1:1000; Cell Signaling Technology), IκBα (#4814, 1:1000; Cell Signaling Technology), NF‐κB p65 (ab16502, 1:500; Abcam), phosphorylated NF‐κB p65 (p‐p65; ab131100, 1:1000; Abcam), NAD(P)H quinone dehydrogenase 1 (NQO1; ab80588, 1:10,000; Abcam), heme oxygenase 1 (HO1; ab52947, 1:2000; Abcam), NOX4 (ab154244, 1:2000; Abcam), and β‐actin (#4970, 1:1000; Cell Signaling Technology). Moreover, goat anti‐rabbit IgG H&L (HRP) (A0208, 1:10,000) and goat anti‐mouse IgG H&L (HRP, 1:10,000) (A0216) were the secondary antibodies used in this assay.

### 2.9 Statistical analysis

GraphPad Prism 8.0 (GraphPad) was used to implement the statistical analyses. The data were expressed as mean ± standard deviation. The multigroup comparison was conducted with one‐way analysis of variance. The statistical significance was deemed when the *p* value was less than .05.

## 3 RESULTS

### 3.1 Luteolin attenuated the effects of CS on inhibiting the body weight and activities of SOD and CAT and promoting MDA levels in COPD model mice

**Figure 1:**
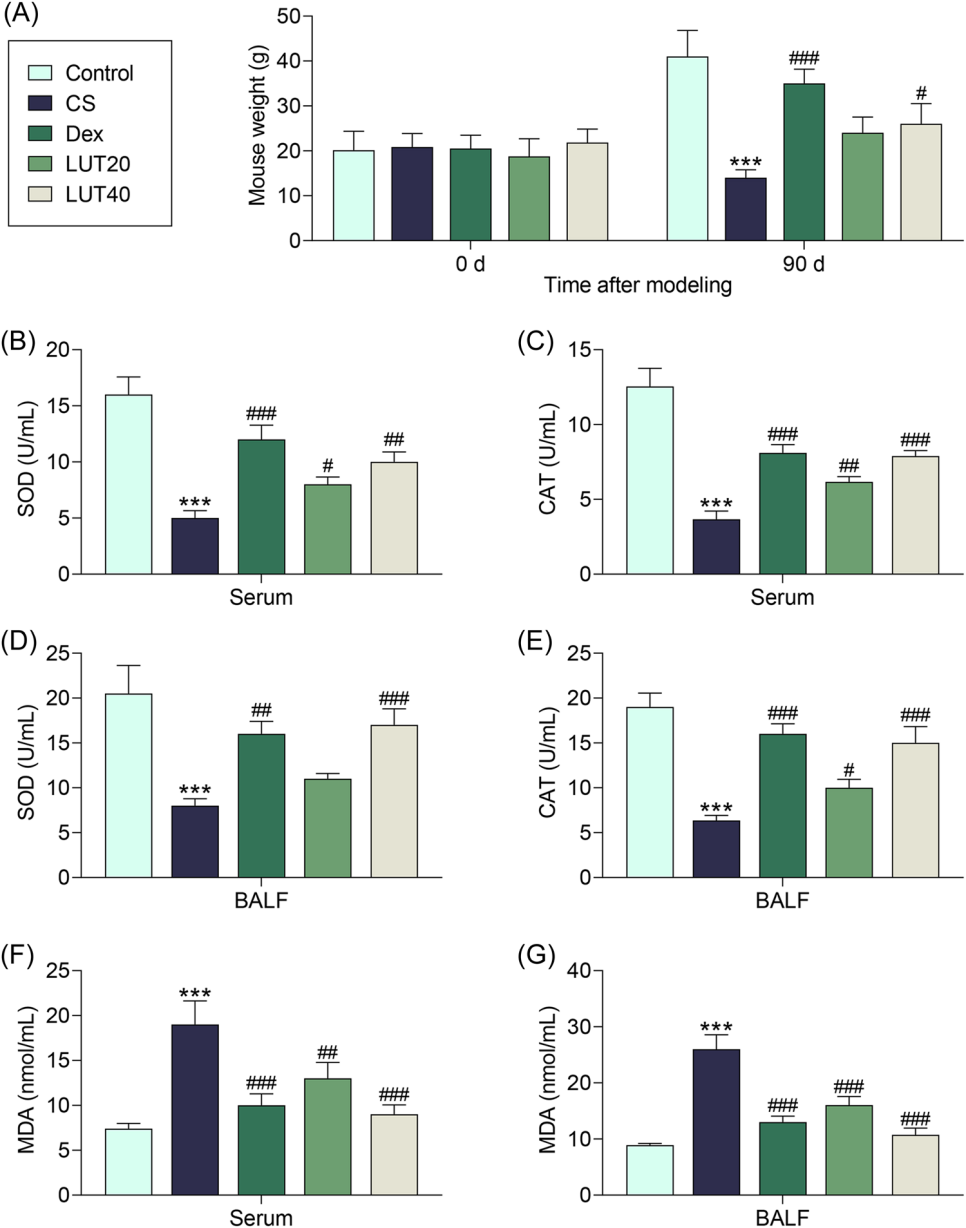
Luteolin reversed the effects of CS on reducing body weight and activities of SOD, and CAT, and increasing MDA levels in mice. (A–G) Mouse model of COPD was constructed by CS exposure. Then the COPD model mice were treated with 2 mg/kg Dex or 20 and 40 mg/kg luteolin for 75 days. (A) The body weight of mice was determined by an electronic balance. (B–G) After the collection of serum and BALF samples, the activities of SOD, CAT, and MDA in the serum and BALF were detected by ELISA (***p < .001 vs. Control; ^#^ *p* < .05, ^##^ *p* < .01, ^###^ *p* < .001 vs. CS). BALF, bronchoalveolar lavage fluid; CAT, catalase; COPD, chronic obstructive pulmonary disease; CS, cigarette smoke; Dex, dexamethasone; LUT, luteolin; MDA, malondialdehyde; SOD, superoxide dismutase.

Considering its verified role in the treatment of COPD, Dex was used as a positive control in our experiment.^38^ The body weight of mice was first calculated, where no significant difference has been reported in the beginning. However, 90 days after the modeling or treatment, the body weight of mice in the CS group was lower than that in the Control group (Figure 1A, *p* < .001). Besides, the body weight of treated mice with Dex and LUT 40 groups increased obviously as compared with that in the CS group (Figure 1A, *p* < .001 and *p* < .05, respectively).

Meanwhile, we assessed the effects of luteolin on the activities of SOD and CAT in mouse serum, with Dex as the positive control. The relevant data were shown in Figure 1B,C. In both of serum and BALF, it was evident that the activities of SOD and CAT were suppressed in the CS group relative to those in the Control group (*p* < .001). Additionally, in the serum of mice, the activity of SOD in Dex and LUT 20/40 groups was elevated as compared with that in the CS group (Figure 1B, *p* < .001, *p* < .05, and *p* < .01, respectively); and the activity of CAT in Dex and LUT 20/40 groups were elevated as compared with that in CS group (Figure 1C, *p* < .001, *p* < .01, and *p* < .001, respectively). The same comparing results were observed in the BALF of mice. Specifically, the activity of SOD in Dex and LUT 40 groups were higher than those in the CS group (Figure 1D, *p* < .01 and *p* < .001, respectively); and the activity of CAT in Dex and LUT 20/40 groups were higher than those in the CS group (Figure 1E, *p* < .001, *p* < .05, and *p* < .001, respectively). These data indicated that luteolin can improve the activities of SOD and CAT in COPD mice, and further ameliorated the antioxidation defense mechanism in COPD mice.

Moreover, the level of MDA in mice of different groups was measured. According to Figure 1F,G, the level of MDA in both serum and BALF was markedly elevated in the CS group (*p* < .001), which was then reduced in the Dex group (*p* < .001) or LUT 20 group (*p* < .01 and *p* < .001, respectively) or LUT 40 groups (*p* < .001).

### 3.2 Luteolin inhibited the inflammation, oxidative stress, and NOX4‐mediated NF‐κB signaling pathway in CS‐treated mice

**Figure 2:**
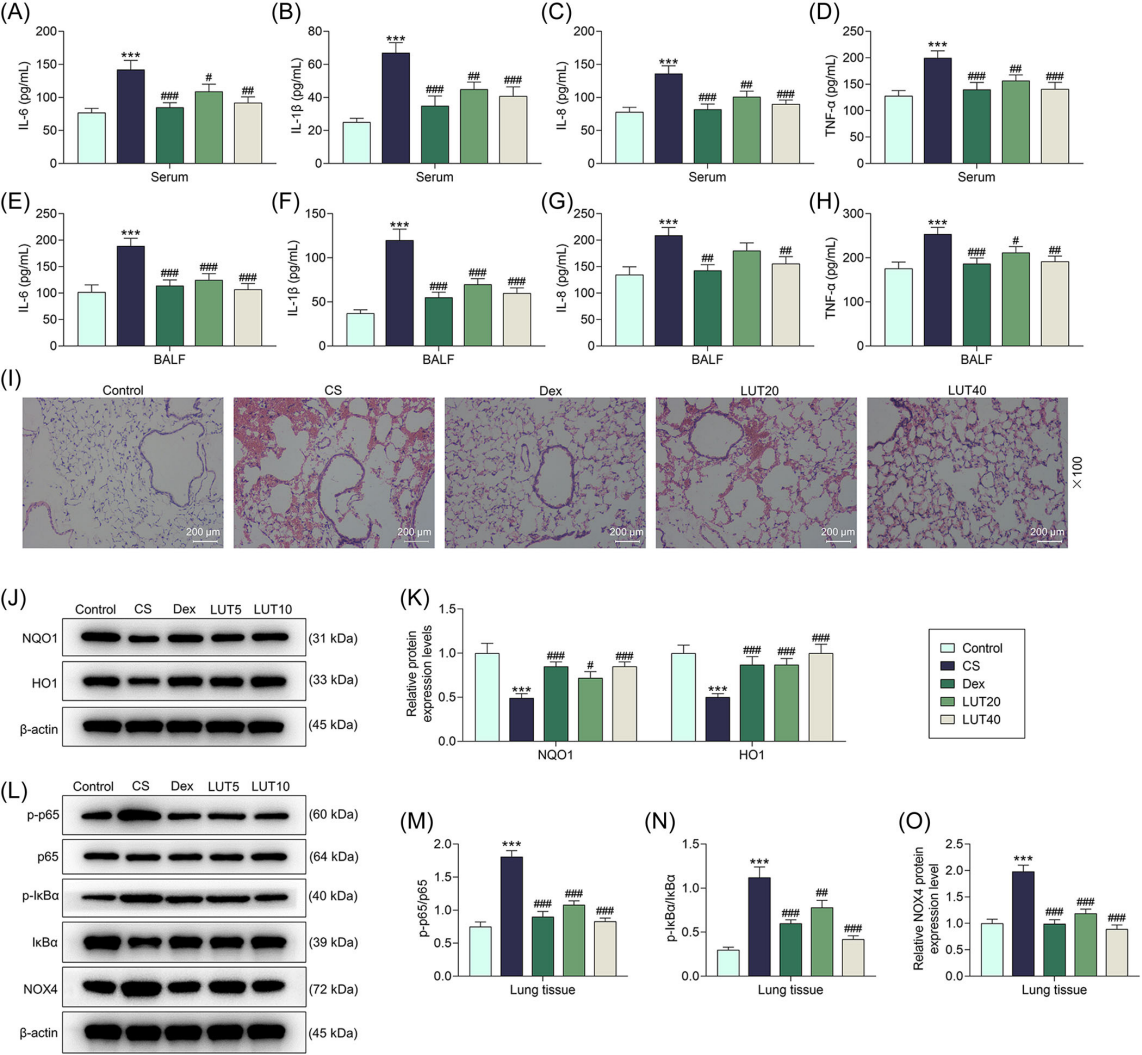
Luteolin attenuated the inflammation and oxidative stress and inactivated the NOX4‐mediated NF‐κB signaling pathway in CS‐treated mice. (A–O) Following the construction of the COPD model by CS, these COPD model mice were treated with either 2 mg/kg Dex or 20 and 40 mg/kg luteolin for 75 days. (A–H) Then serum and BALF samples were subsequently collected, and an enzyme‐linked immunosorbent assay was used to determine the levels of IL‐6, IL‐1β, IL‐8, and TNF‐α in the serum and BALF. (I) Also, the lung tissue of mice was collected and observed by H&E staining under an optical microscope (magnification: ×100, scale bar = 200 μm). (J, K) The expressions of oxidative stress‐related proteins NQO1 and HO1 were quantified by Western blot assay. β‐Actin was used as the housekeeping control. (L–O) Western blot assay was also employed to quantify the levels of NOX4‐mediated NF‐κB signaling‐related proteins. β‐Actin was used as an internal control (***p < .001 vs. Control; ^#^ *p* < .05, ^##^ *p* < .01, ^###^ *p* < .001 vs. CS). BALF, bronchoalveolar lavage fluid; COPD, chronic obstructive pulmonary disease; CS, cigarette smoke; Dex, dexamethasone; H&E, hematoxylin‐eosin; HO1, heme oxygenase 1; IL, interleukin; LUT, luteolin; NF‐κB, nuclear factor kappa‐B; NOX4, NADPH oxidase 4; NQO1, NAD(P)H quinone dehydrogenase 1; p‐IκB, phosphorylated‐I‐kappa‐B; p‐p65, phosphorylated‐p65; TNF‐α, tumor necrosis factor‐α.

The levels of inflammation‐related cytokines, including IL‐1β, IL‐6, TNF‐α, and IL‐8, in both serum and BALF, were examined by ELISA. The results suggested that the exposure to CS led to upregulated levels of IL‐1β, IL‐6, TNF‐α, and IL‐8 (Figure 2A–H, *p* < .001), while Dex abrogated the CS‐induced upregulation of these inflammation‐related cytokines (Figure 2A–H, *p* < .001 for IL‐6 and IL‐1β and TNF‐α, *p* < .001 or *p* < .01 for IL‐8), and luteolin (at 20 and 40 mg/kg) also inhibited the CS‐induced upregulation of these inflammation‐related cytokines (Figure 2A–H, *p* < .05, *p* < .01, or *p* < .001 for IL‐6 and TNF‐α, *p* < .01 or *p* < .001 for IL‐1β and IL‐8). The data of the H&E staining assay indicated that after the treatment with CS, the alveolar destruction was significantly aggravated, with some critical injuries in the lung tissue of COPD model mice. On the contrary, the intervention of luteolin (at 20 and 40 mg/kg) and Dex can alleviate the injury of lung tissue (Figure 2I).

Western blot assay was further performed to quantify the expressions of oxidative stress‐related proteins, including NQO1 and HO1. The results proved that NQO1 and HO1 expressions were inhibited in CS‐treated mice when compared with those in the Control group (Figure 2J,K, *p* < .001). Meanwhile, Dex and luteolin reversed the inhibiting effects of CS on the expressions of NQO1 and HO1 in COPD model mice (Figure 2J,K, *p* < .001, *p* < .05, and *p* < .001 for NQO1, *p* < .001 for HO1). Then, NF‐κB signaling pathway‐related proteins (p‐p65, p65, p‐IκB, and IκB) in tissue samples were quantitated by Western blot assay as well. In accordance with the results, the ratios of p‐p65/p65 and p‐IκB/IκB were increased following the exposure to CS (Figure 2L–N, *p* < .001), but were then decreased with the treatment of Dex (*p* < .001) and luteolin (*p* < .001 for p‐p65/p65, *p* < .01 and *p* < .001 for p‐IκB/IκB) in model mice (Figure 2L–N).

Based on the analysis by AutoDock software, luteolin was predicted to bind with NOX4 (Supporting Information: Figure 1A). Therefore, we further quantified the expression of NOX4 in the lung tissues of mice. As illustrated in Figure 2L,O, NOX4 level was upregulated by CS exposure (*p* < .001), and then downregulated by Dex and luteolin treatment (*p* < .001). It can be concluded that luteolin inhibited inflammation, oxidative stress, and NOX4‐mediated NF‐κB signaling pathway in CS‐treated mice.

### 3.3 Luteolin alleviated CS‐induced inflammation, oxidative stress, and NOX4‐mediated NF‐κB signaling pathway activation in CS‐treated cells

To figure out the effect of luteolin on human alveolar epithelial cells, human A549 cells received the intervention of luteolin, and cell viability was determined. The data of the CCK‐8 assay were shown in Supporting Information: Figure 1B. It can be noticed that cell viability was remarkably decreased after the treatment of 400 μM luteolin for 48 h (*p* < .05). Therefore, luteolin with the concentrations of 100 and 200 μM was chosen for subsequent assays.

**Figure 3:**
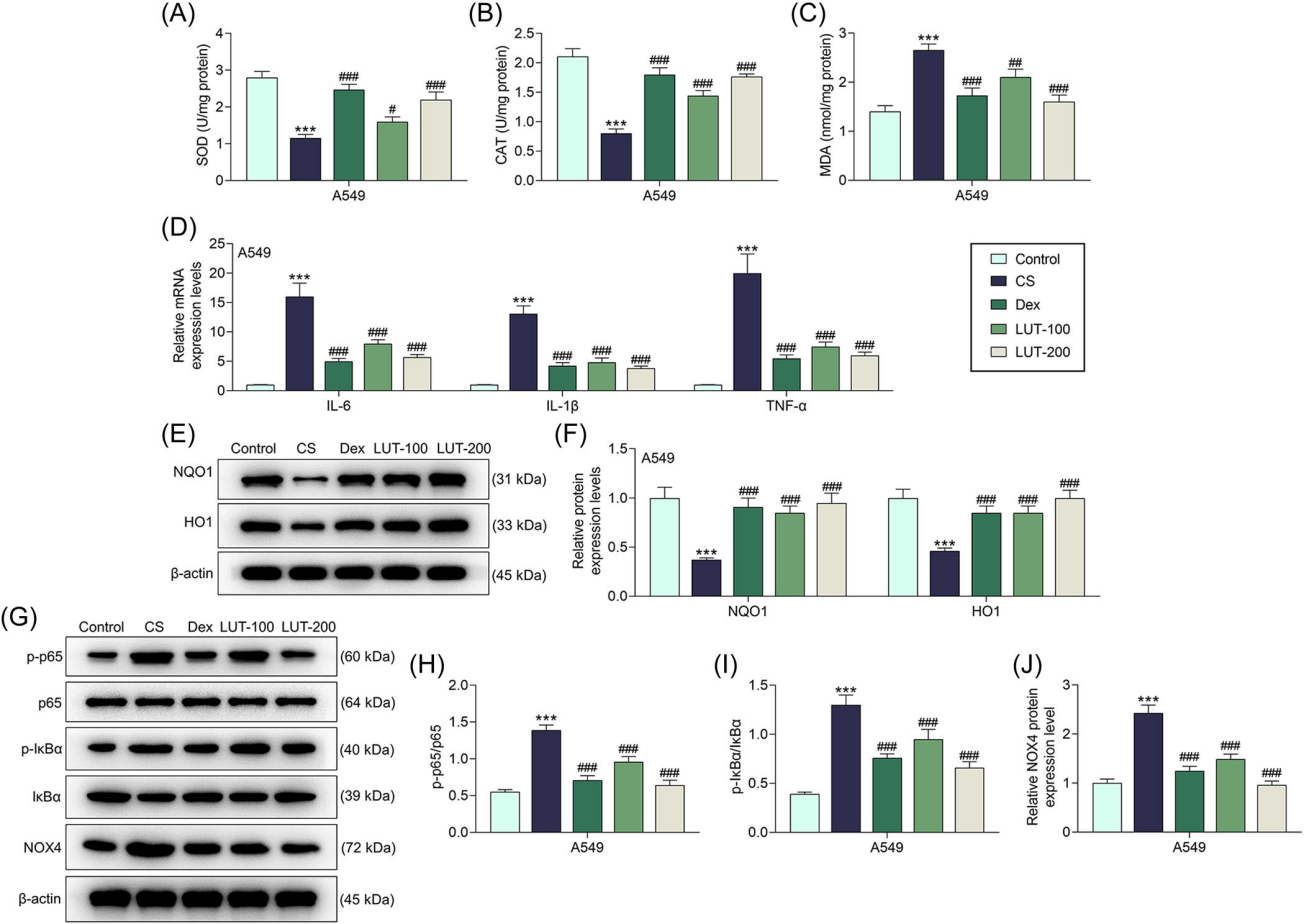
Luteolin alleviated CS‐induced inflammation, oxidative stress, and NOX4‐mediated NF‐κB signaling pathway activation in CS‐treated cells. (A–J) A549 cells were exposed to 1% CSE for 24 h and then treated with Dex (1 μΜ) or luteolin (100 or 200 μΜ for 24 h). (A–C) Subsequently, the activities of SOD, CAT, and MDA were measured using ELISA. (D) The levels of proinflammatory factors (IL‐6, IL‐1β, TNF‐α) were quantified via qRT‐PCR. β‐Actin was used as the internal control. (E–J) Western blot assay was used to calculate the expressions of NQO1, HO1, and NOX4‐mediated NF‐κB signaling pathway‐related proteins. β‐Actin was employed as the internal control (***p < .001 vs. Control; ^#^ *p* < .05, ^##^ *p* < .01, ^###^ *p* < .001 vs. CS). CAT, catalase; CS, cigarette smoke; CSE, cigarette smoke extract; Dex, dexamethasone; HO1, heme oxygenase 1; IL, interleukin; LUT, luteolin; MDA, malondialdehyde; NF‐κB, nuclear factor kappa‐B; NOX4, NADPH oxidase 4; NQO1, NAD(P)H quinone dehydrogenase 1; p‐IκB, phosphorylated‐I‐kappa‐B; p‐p65, phosphorylated‐p65; qRT‐PCR, quantitative real‐time PCR; SOD, superoxide dismutase; TNF‐α, tumor necrosis factor‐α.

Then, the factors related to inflammation, oxidative stress, and NF‐κB signaling pathway were measured after CSE exposure (CS‐treated cells) or Dex and luteolin treatment. As depicted in Figure 3A–C, the activities of SOD and CAT dwindled and the level of MDA was augmented in CS‐treated cells (*p* < .001). Such abnormal levels of SOD, CAT, and MDA were then restored by Dex and luteolin (*p* < .001 for the Dex group, *p* < .05, *p* < .001, and *p* < .01 for LUT 100 group, *p* < .001 for LUT 200 group).

qRT‐PCR was also exploited to calculate the expressions of proinflammatory factors including IL‐6, IL‐1β, and TNF‐α. Correspondingly, it was demonstrated that the levels of these proinflammatory factors were upregulated in CS‐treated cells (Figure 3D, *p* < .001), while Dex and luteolin can offset the effects of CS on these proinflammatory factors (Figure 3D, *p* < .001).

Likewise, the expressions of oxidative stress‐related proteins and NOX4‐mediated NF‐κB signaling pathway‐related proteins were detected by Western blot assay. The results confirmed that NQO1 and HO1 expressions were downregulated (*p* < .001), yet the ratios of p‐p65/p65 (*p* < .001) and p‐IκB/IκB (*p* < .001) and expression of NOX4 (*p* < .001) were upregulated in CS‐treated A549 cells (Figure 3E–J). However, after Dex and luteolin treatment, the expressions of NQO1 (*p* < .001) and HO1 (*p* < .001) were increased yet the ratios of p‐p65/p65 (*p* < .001) and p‐IκB/IκB (*p* < .001) and expression of NOX4 (*p* < .001) were decreased in CS‐treated cells (Figure 3E–J, *p* < .001). These results thus indicated that luteolin can alleviate CS‐induced inflammation, oxidative stress, and NOX4‐mediated NF‐κB signaling pathway activation in CS‐treated cells.

### 3.4 Luteolin ameliorated CS‐induced inflammation, oxidative stress, and NF‐κB signaling pathway activation in CS‐treated cells by regulating NOX4

**Figure 4:**
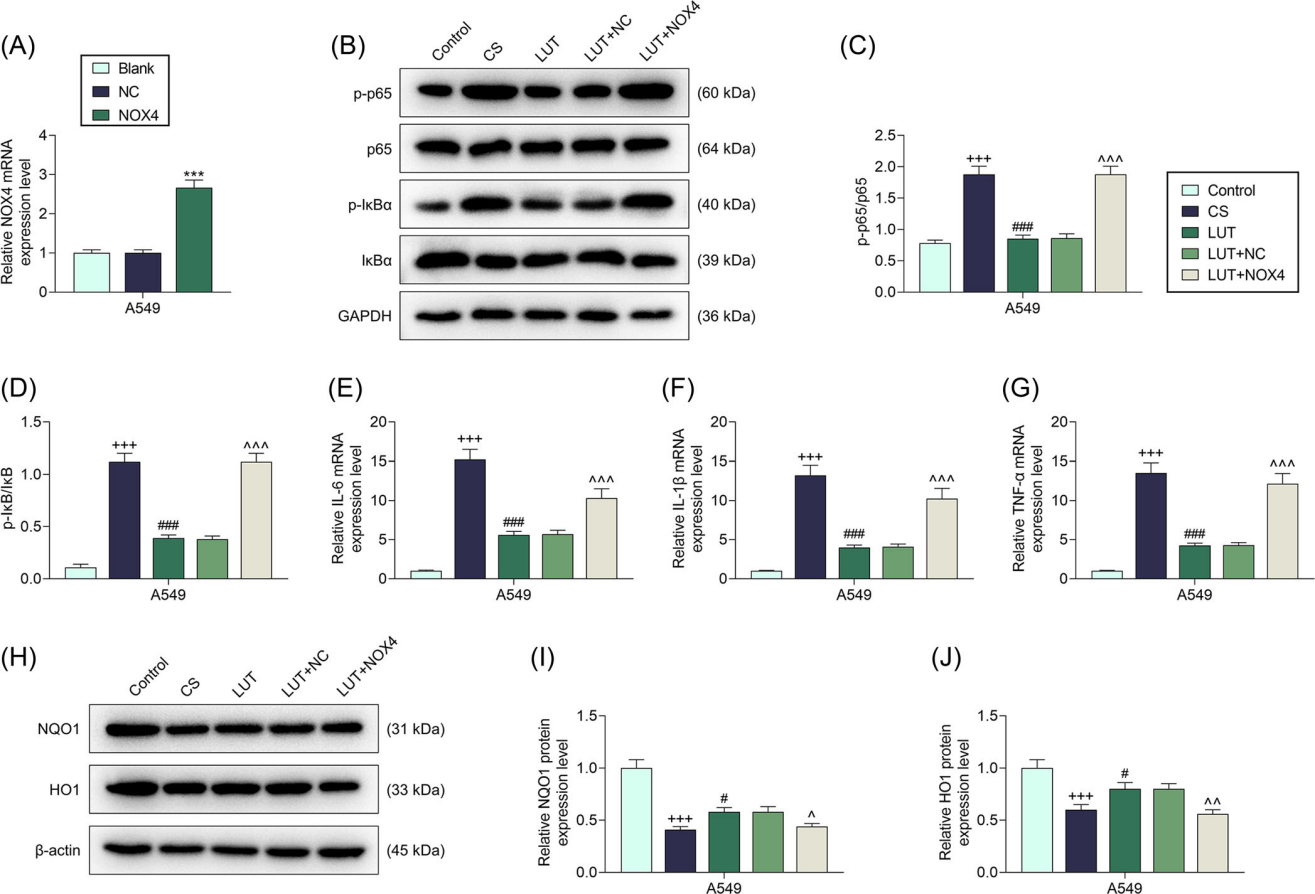
Luteolin mitigated CS‐induced inflammation, oxidative stress, and NF‐κB signaling pathway activation in CS‐treated cells by regulating NOX4. (A) After A549 cells were transfected with NOX4 overexpression plasmids, the expression of NOX4 in cells was evaluated using qRT‐PCR. β‐Actin was used as the internal control. (B–D) After the transfected cells were exposed to 1% CSE for 24 h and then treated with 200 μΜ luteolin for 24 h, the expressions of NF‐κB signaling pathway‐related proteins were detected using Western blot, with β‐actin as the internal control. (E–G) The expressions of proinflammatory factors (IL‐6, IL‐1β, TNF‐α) in these cells were calculated via qRT‐PCR. β‐Actin was used as the internal control. (H–J) The expressions of oxidative stress‐related proteins (NQO1 and HO1) in these cells with various interventions were quantified using Western blot assay. β‐Actin served as the internal control (***p < .001 vs. NC; ^+++^ *p* < .001 vs. Control; ^#^ *p* < .05, ^###^ *p* < .001 vs. CS, ^^^ *p* < .05, ^^^^ *p* < .01, ^^^^^ *p* < .001 vs. LUT + NC). CS, cigarette smoke; CSE, cigarette smoke extract; HO1, heme oxygenase 1; IL, interleukin; LUT, luteolin; NF‐κB, nuclear factor kappa‐B; NOX4, NADPH oxidase 4; NQO1, NAD(P)H quinone dehydrogenase 1; p‐IκB, phosphorylated‐I‐kappa‐B; p‐p65, phosphorylated‐p65; qRT‐PCR, quantitative real‐time PCR; TNF‐α, tumor necrosis factor‐α.

To further verify whether luteolin impacts CS‐induced A549 cells by targeting NOX4, NOX4 was overexpressed in A549 cells via the transfection of NOX4 overexpression plasmids (Figure 4A, *p* < .001). Then, the levels of factors related to inflammation, oxidative stress, and the NF‐κB signaling pathway were further determined. As exhibited in Figure 4B–D, the ratios of p‐p65/p65 (*p* < .001) and p‐IκB/IκB (*p* < .001) were upregulated in CS‐treated cells but were then downregulated by 200 μM luteolin (*p* < .001). Besides, NOX4 overexpression reversed the effects of luteolin on the ratios of p‐p65/p65 and p‐IκB/IκB in CS‐treated cells (*p* < .001).

Next, it was visible that the levels of inflammation‐related factors, including IL‐6 (*p* < .001), IL‐1β (*p* < .001), and TNF‐α (*p* < .001), was increased by CS, all of which was then decreased by 200 μM luteolin in CS‐treated A549 cells (Figure 4E–G, *p* < .001). Similarly, NOX4 overexpression reversed the effects of luteolin on the inflammation‐related factors in CS‐treated cells (Figure 4E–G, *p* < .001). Also, the levels of oxidative stress‐related proteins NQO1 (*p* < .001) and HO1 (*p* < .001) were discovered to be downregulated by CS, which were then upregulated by 200 μM luteolin in CS‐treated cells (Figure 4H–J, *p* < .05). In addition, NOX4 overexpression counteracted the roles of luteolin in the levels of oxidative stress‐related proteins in CS‐treated cells (Figure 4H–J, *p* < .05, *p* < .01). These data reflected that luteolin alleviated CS‐induced inflammation, oxidative stress, and the activation of NF‐κB signaling pathway in CS‐treated cells by regulating NOX4.

## 4 DISCUSSION

COPD is a global healthcare problem with a steadily growing incidence that has been studied for more than 200 years, and CS‐induced mitochondrial damage has been proven to be the major cause of COPD.^39^, ^40^ Mitochondrial oxidative stress may be particularly important in COPD. It has been reported that increased oxidative stress in the lung is a major driver of the disease through multiple interacting molecular mechanisms.^41^ In addition, inflammation has also been highlighted as the main pathogenic factor causing COPD.^42^ Specifically, the extrapulmonary manifestation of COPD is mostly chronic systemic inflammation.^43^ Moreover, the symptoms such as excessive mucus and airway inflammation caused by CS are the causes of COPD, and carbocisteine can be used to remove mucus in COPD.^44^ In this study, our results revealed that luteolin inhibited inflammation and oxidative stress in COPD via NOX4‐mediated NF‐κB signaling pathway inactivation, as evidenced by decreased inflammation factors and oxidative stress factors as well as increased antioxidant factors in CS‐induced COPD mice and A549 cells.

Luteolin has been applied to treat assorted diseases in clinical practice.^45^ Luteolin can protect intestinal mucositis with its antioxidant and anti‐inflammatory properties.^46^ In a previous study, luteolin has been used in the treatment of COPD, the mechanism of which is associated with the regulation of epidermal growth factor receptor, matrix metallopeptidase 9, and so forth.^47^ Furthermore, luteolin was proven to promote mucociliary clearance in COPD.^20^ As an angiotensin receptor blocker, Dex is widely used to treat COPD, due to its abilities to improve oxidative stress activity, attenuating inflammation, and repressing the levels of NF‐κB pathway‐related proteins.^48^ In our study, we found that luteolin had a similar function as Dex on CS‐exposed mice. To be specific, luteolin increased the mouse weight and improved lung tissue damage in CS‐exposed mice. Moreover, luteolin decreased the inflammation factor (IL‐6, IL‐1β, IL‐8, TNF‐α) levels and oxidative stress factor (MDA) activity, and increased the antioxidant factor activities (SOD and CAT) in CS‐exposed mice and A549 cells. Nevertheless, the specific action mechanism of luteolin in these models remained unclear.

Anti‐inflammation and antioxidants are important therapeutic strategies to inhibit COPD. As antioxidant enzymes, SOD can catalyze superoxide to produce oxygen and hydrogen peroxide, while CAT can be used in neutralizing hydrogen peroxide by converting it into oxygen molecules and water.^11^ MDA is a signal and production of lipid peroxidation which is used to evaluate the occurrence of lipid peroxidation.^49^ Furthermore, NQO‐1 and HO‐1 constitute an important antioxidant defense signal pathway. In other words, the antioxidant effect can be achieved via upregulating the levels of NQO‐1 and HO‐1.^50^, ^51^ Moreover, IL‐6, IL‐1β, and TNF‐α have been extensively researched in inflammatory diseases, the decrease of which has been suggested to inhibit inflammation.^51^, ^52^ It has been documented that luteolin can exert antioxidant and anti‐inflammatory effects possibly through inhibiting the expressions of IL‐6, IL‐1β, TNF‐α, and MDA, and promoting the activities of SOD and CAT.^53^ Our results showed that luteolin can attenuate the effects of CS on elevating the level of MDA, promoting the expressions of IL‐1β, IL‐6, IL‐8, and TNF‐α, repressing the expressions of NQO‐1 and HO‐1, and diminishing the activities of SOD and CAT. It indicated that luteolin impacted COPD by inhibiting oxidative stress and inflammatory responses.

NOX4, one of the major sources of ROS, has multiple biological activities in cancer, inflammation, bacterial dysbiosis, metabolism, and so on.^10^, ^54^, ^55^, ^56^, ^57^ More importantly, luteolin can suppress the proliferation of smooth muscle cells by reducing NOX4 production.^12^ Similarly, in this research, we proved that luteolin can bind with and target NOX4. It has been underlined that NOX4 expression is not only higher in COPD model epithelial cells and small airway smooth muscle cells, but also positively correlated with the severity of chronic obstructive pulmonary airflow.^11^ Consistently, in our current research, the presence of NOX4 was also discovered in COPD model mice and cells, and its level can be downregulated by luteolin, indicating that the effects of luteolin on inhibiting oxidative stress and inflammatory responses in COPD might be achieved by downregulating NOX4 level.

Previous research has unveiled that luteolin ameliorates TNF‐α‐induced oxidative stress and inflammation in HUVECs by suppressing NOX4/ROS‐NF‐κB pathway.^28^ The suppression of the NOX4‐mediated NF‐κB signaling pathway is also proven to have an anti‐inflammatory effect in macrophages.^58^ Considering the role of NF‐κB signaling in the regulation of the expressions of inflammation‐related genes, it has been recognized as a typical proinflammatory signaling.^22^ An existing study has revealed that luteolin can protect against diabetic cardiomyopathy by regulating NF‐κB signaling‐mediated inflammation and activating antioxidant responses.^21^ It has been reported that alantolactone suppresses inflammation, apoptosis, and oxidative stress in CS‐induced human bronchial epithelial cells through activation of Nrf2/HO‐1 and inhibition of the NF‐κB pathway.^59^ Also, CS has already been proven to promote the activation of NF‐κB signaling by inducing the autophagy of the pulmonary macrophages.^60^ In this research, we discovered that exposure to CS led to the activated NF‐κB signaling in the COPD model both in vitro and in vivo. Meanwhile, under the regulation of luteolin, which had similar regulatory effects as Dex, NF‐κB signaling was notably inhibited. Furthermore, the effects of luteolin on CS‐induced inflammation, oxidative stress, and NF‐κB signaling were further reversed by NOX4 overexpression. These data revealed that the effects of luteolin on COPD model mice and cells were realized by targeting NOX4.

Collectively, our results confirm that luteolin can alleviate inflammation and oxidative stress in COPD via NOX4‐mediated NF‐κB signaling inhibition, which provides a theoretical basis for the application of luteolin in the treatment of COPD.

## AUTHOR CONTRIBUTIONS

**Mingfei Li**: Writing—review and editing (equal); conceptualization (lead); writing—original draft (lead); formal analysis (lead). **Huifang Wang**: Conceptualization (lead); writing—original draft (lead); formal analysis (lead); writing—review and editing (equal). **Yun Lu**: Software (lead); writing—review and editing (equal); methodology (lead). **Jinwen Cai**: Conceptualization (supporting); writing—original draft (supporting); writing—review and editing (equal); project administration.

## CONFLICT OF INTEREST STATEMENT

The authors declare no conflict of interest.

## ETHICS STATEMENT

All animal experiments were conducted in accordance with the China Council on Animal Care and Use. This study has obtained the approval from the Committee of Experimental Animals of Nanfang Hospital (serial number: HZYY‐202102201), and the animal experiments in our study were conducted in Nanfang Hospital. Every effort was dedicated to minimizing the pain and discomfort to the experimental animals.

## Supporting information

Supporting information.

## Data Availability

The analyzed data sets generated during the study are available from the corresponding author on reasonable request.
