# Supplementary figures and images for: Luteolin suppresses inflammation and oxidative stress in chronic obstructive pulmonary disease through inhibition of the NOX4‐mediated NF‐κB signaling pathway
Source: Immun Inflamm Dis. 2023 Apr 27;11(4):e820. doi: 10.1002/iid3.820 (PMC10134768; doi:10.1002/iid3.820)

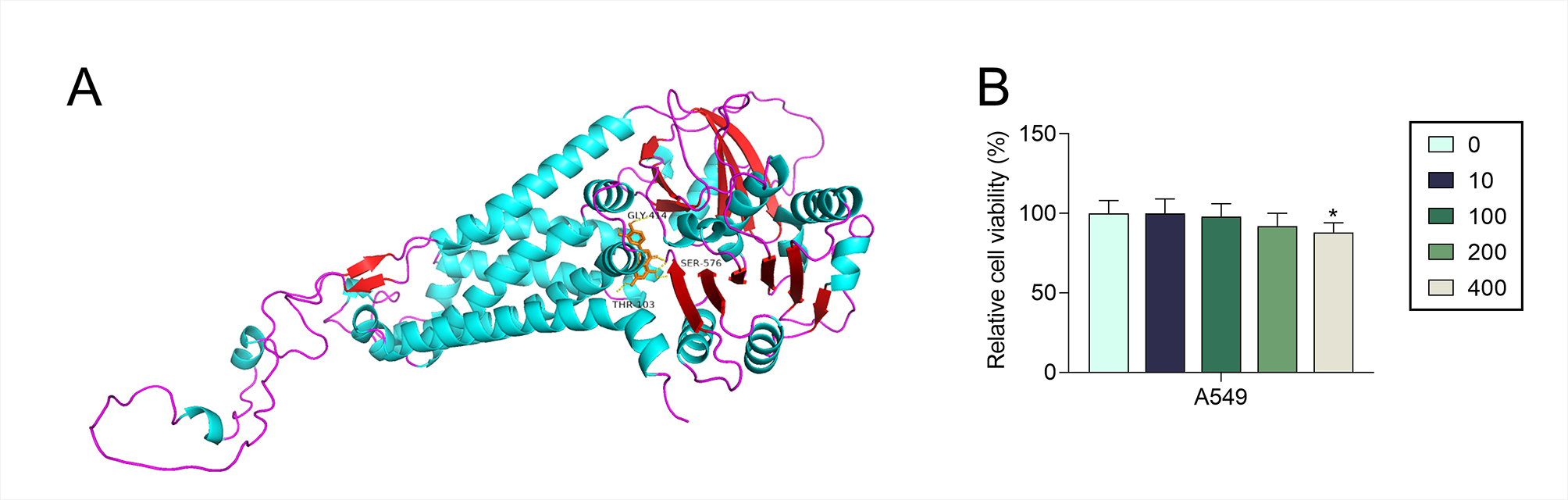

Supplement: Supplementary file 1 — Supporting information. [file IID3-11-e820-s001.tif]
